# Influence of cytoskeleton organization on recombinant protein expression by CHO cells

**DOI:** 10.1002/bit.27277

**Published:** 2020-02-23

**Authors:** Lucille Pourcel, Flavien Buron, Ghislaine Arib, Valérie Le Fourn, Alexandre Regamey, Iris Bodenmann, Pierre‐Alain Girod, Nicolas Mermod

**Affiliations:** ^1^ Department of Fundamental Microbiology, Institute of Biotechnology University of Lausanne Lausanne Switzerland; ^2^ Selexis SA Geneva Switzerland

**Keywords:** actin polymerization, CHO cells, cytoskeleton regulation, recombinant protein production

## Abstract

In this study, we assessed the importance of cytoskeleton organization in the mammalian cells used to produce therapeutic proteins. Two cytoskeletal genes, Actin alpha cardiac muscle 1 (*ACTC1*) and a guanosine triphosphate GTPase‐activating protein (*TAGAP*), were found to be upregulated in highly productive therapeutic protein‐expressing Chinese hamster ovary (CHO) cells selected by the deprivation of vitamin B5. We report here that the overexpression of the ACTC1 protein was able to improve significantly recombinant therapeutic production, as well as to decrease the levels of toxic lactate metabolic by‐products. ACTC1 overexpression was accompanied by altered as well as decreased polymerized actin, which was associated with high protein production by CHO cell cultured in suspension. We suggest that the depolymerization of actin and the possible modulation of integrin signaling, as well as changes in basal metabolism, may be driving the increase of protein secretion by CHO cells.

## Graphical abstract

Pourcel et al. observed that several cytoskeletal genes are upregulated in highly productive CHO cell clones. Actin alpha overexpression yielded altered cytoskeletal organization, which was accompanied by improved recombinant therapeutic production as well as decreased levels of toxic lactate metabolic by‐products. Cells spontaneously displaying low actin polymerization yielded higher protein production in suspension cultures, highlighting the importance of the cytoskeleton for the efficient production of easy‐ as well as difficult‐to‐express therapeutic proteins.

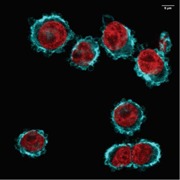

## 1 INTRODUCTION

Improving recombinant therapeutic protein manufacturing using mammalian Chinese hamster ovary (CHO) cell hosts is a major challenge for biopharmaceutical companies. Thus, many research are currently ongoing to unravel and overcome the remaining unknown metabolic bottlenecks of these cells for high‐level protein production. We previously described how the deprivation of vitamin B5 in CHO cell culture medium, coupled to the cotransfection of an expression vector for a vitamin B5 transport protein with the gene of interest, allowed the identification of cell variants that are capable of expressing at very high levels recombinant therapeutic proteins (Pourcel et al., 2020). However, the cellular properties that allow very high‐level recombinant protein secretion remain unknown.

In this study, we wished to identify specific gene expression alterations that accompany efficient therapeutic protein expression, with the hope to construct CHO cell line derivatives that would be permanently more efficient for production. Among these alterations, two genes involved in cytoskeleton organization were significantly induced after vitamin B5 selection, namely actin alpha cardiac muscle 1 (ACTC1) and a guanosine triphosphate GTPase‐activating protein (TAGAP). ACTC1 is the major protein of the cardiac sarcomere thin filaments, which are responsible for the muscle contraction function of the heart. Consistently, ACTC1 deficiency has been mainly linked to heart diseases (Debold et al., 2010; Wang et al., 2016). TAGAP is a signaling protein that was involved in thymocyte loss of adhesion and thymocyte and T cells cytoskeleton reorganization (Connelly et al., 2014; Duke‐Cohan et al., 2018). Alterations of the TAGAP gene have been associated with various autoimmune diseases (Eyre et al., 2010).

Cytoskeleton consists of a network of actin microfilaments, microtubule, and intermediate filaments required for multiple cellular processes, such as cell shape and resistance to mechanical deformation (Mays, Beck, & Nelson, 1994), protein synthesis (Hudder, Nathanson, & Deutscher, 2003), protein transport and secretion (Paavilainen, Bertling, Falck, & Lappalainen, 2004; Stamnes, 2002), association of cellular components (Knull & Walsh, 1992), and metabolic channeling (Aon & Cortassa, 2002). Moreover, an increase in monoclonal antibody production was correlated with a significant increase in cytoskeletal proteins such as actin, tubulin, or the actinin‐binding cofilin (Dinnis et al., 2006). Recent studies have shown that suspension CHO cells have evolved from adherent cells by disruption of the extracellular attachment matrix accompanied with major changes in the cytoskeleton, such as increased actin filament expression, which is required for proper interaction with integrins, resistance to shear stress, and cell proliferation in suspension (Walther, Whitfield, & James, 2016). Therefore, cytoskeleton organization and modulation of actin filament levels may impact suspension cell fitness and recombinant protein expression, from messenger RNA (mRNA) translation to protein secretion.

Here, we studied the effect of ACTC1 and TAGAP on recombinant protein expression. We show that ACTC1 overexpression improves the production of easy‐to‐express and hard to express recombinant proteins. We further identify a link between an overall decrease of actin polymerization and an increase in recombinant protein secretion, which we hypothesize is mediated by the cytoskeleton structure and/or stability.

## 2 MATERIALS AND METHODS

### 2.1 DNA vector constructs

Genomic and complementary DNA (cDNA) sequences of the ACTC1 and TAGAP genes were determined after alignment to the homologous genes in mice using NCBI BLAST software. Transcript sequence RNA‐seq analysis was performed on Selexis SA CHO‐K1 cells (CHO‐M). The cDNA libraries were generated by reverse transcription from 1 µg total RNA isolated from 10^6^ CHO‐M cells (NucleoSpin™ RNA kit; Macherey‐Nagel) using the GoScript Reverse transcription System (Promega). The ACTC1 and TAGAP coding sequences (CDS) were cloned into the *pBSK_ITR_BT*+*_EGFP_X29_ITR* transposable expression vector (Le Fourn, Girod, Buceta, Regamey, & Mermod, 2014), yielding the pBSK‐ACTC1 and pBSK‐TAGAP expression vectors. The expression cassette is flanked by the inverted terminal sequences of the piggyBac transposon. The blasticidin vector (pBlast) contains the blasticidin resistance gene under the control of the SV40 promoter originated from pRc/RSVplasmid (Invitrogen/Life Technologies).

### 2.2 Cell culture and stable transfections

CHO‐K1 cells were maintained in suspension culture in SFM4CHO Hyclone serum‐free medium (SFM; Thermo Fisher Scientific™) supplemented with l‐glutamine (PAA, Austria) and HT supplement (Gibco, Invitrogen Life Sciences) at 37°C, 5% CO_2_ in humidified air. Other cell media used for these experiments is the Deficient BalanCD CHO Growth A (B‐CDmin; Irvine Scientific), supplemented with vitamin B1 (thiamine hydrochloride; Sigma‐Aldrich), vitamin B5 (Calcium DL‐Pantothenate; TCI) and vitamin H (Biotin; Sigma‐Aldrich). CHO cells were transfected with pBSK‐ACTC1 or TAGAP, pBlast, and pCS2‐U5‐PBU3 IgG1‐Hc or IgG1‐Lc expression vectors by electroporation according to the manufacturer's recommendations (Neon devices, Invitrogen). Original immunoglobulin G (IgG)‐producing stable cell lines were generated by culturing transfected cells in the SFM4CHO media complemented with 7.5 µg/ml of blasticidin for 3 weeks, followed by the isolation of monoclonal cell populations using the ClonePix™ FL Imager from Molecular Devices.

Cell pool populations expressing the IgG and ACTC1 and/or TAGAP were selected for blasticidin resistance as follow: Cells were seeded in SFM4CHO media supplemented with 10 µg/ml blasticidin for 2 weeks, then cultured into wells containing nonsupplemented culture medium for 5 days, and then transferred into 50 ml spin tubes.

Selection based on vitamin B5 deprivation was performed by culturing the cells cotransfected with the vitamin B5 transporter SLC5A6 expression vector in a chemically defined medium with a low concentration of vitamin B5 (B5‐deprived BalanCD CHO‐M Growth A supplemented with 2.5 nM vitamin B5), as described previously (Pourcel et al., 2020).

### 2.3 Analyses of stable cell pools and cell lines

Fed‐batch performance evaluation, IgG cell surface staining, IgG cell secretion assay, and vitamin B5 metabolite quantification, were performed as previously described (Pourcel et al., 2020). Briefly, IgG secretion performances in fed‐batch culture were performed as previously reported (Le Fourn et al., 2014). The assay of cell surface IgG was as reported previously (Brezinsky et al., 2003), and cell pools secreting high levels of recombinant IgG protein were subcloned using ClonePix™ FL Imager from Molecular Devices. For vitamin B5 metabolite quantification, cell pellets were extracted with 1 ml of cold MeOH:H_2_O (4:1, vol/vol) solvent mixture, then probe‐sonicated. The supernatant obtained after 1 hr incubation at −20°C, followed by 15 min centrifugation at 13,000 rpm at 4°C were collected and evaporated to dryness then reconstituted in 100 µl MeOH:water (4:1) and injected into the liquid chromatography–mass spectrometry (LC–MS) system. The protein pellets were evaporated and lysed in 20 mM Tris‐HCl (pH 7.5), 4 M guanidine hydrochloride, 150 mM NaCl, 1 mM Na2EDTA, 1 mM egtazic acid, 1% Triton, 2.5 mM sodium pyrophosphate, 1 mM β‐glycerophosphate, 1 mM Na_3_VO_4_, 1 µg/ml leupeptin using brief probe‐sonication. Extracted samples were analyzed by hydrophilic interaction liquid chromatography–high resolution mass spectrometry (HRMS) in negative ionization modes using a Q‐Exactive instrument (Thermo Fisher Scientific) operating at mass resolving power of 70,000 full width half maximum. Raw LC–HRMS data were processed using the Thermo Fisher Scientific software (Xcalibur 4.0 QuanBrowser; Thermo Fisher Scientific). Metabolite quantification was performed using external calibration curves.

### 2.4 RNA RT‐PCR and sequencing RNA‐seq analysis

For RNA reverse transcription and real‐time quantitative polymerase chain reaction (RT‐qPCR) analysis, total RNA was extracted from 10^6^ cells and reverse‐transcribed into cDNA using polyT primers. Transcripts accumulation was quantified by qPCR using the SYBR Green‐Taq polymerase kit from Eurogentec Inc, and ABI Prism 7700 PCR machine (Applied Biosystems). Transcript levels were normalized to that of the GAPDH housekeeping gene. RNA‐seq analysis of the B5‐ and puromycin‐selected CHO cell was as previously described (Pourcel et al., 2020).

Briefly, total RNA was extracted from (a) parental CHO cells, (b) CHO cell lines expressing the interferon β and the B5 transporter SLC5A6 expression vectors subjected to B5 deprivation/puromycin selection or puromycin selection only, (c) CHO cell pools expressing the trastuzumab and SLC5A6 expression vectors selected as previously with B5 deprivation/puromycin selection or puromycin selection only. cDNA was obtained from 0.5 to 1 µg of total RNA using the Illumina TruSeq stranded mRNA‐seq reagents (Illumina). The RNA‐seq library 100 nucleotides‐paired end was sequenced on the Illumina HiSeq 2500. Reads were mapped to the CHO‐K1 transcriptome (RefSeq, 2014).

### 2.5 Protein sample preparation and immunoblotting

Total actin content was evaluated as follows. Protein extraction was performed from 10^7^ cells washed in phosphate‐buffered saline (PBS), after which the cell pellet was resuspended in radioimmunoprecipitation assay lysis buffer (150 Mm NaCl, 50 mM Tris‐HCl [pH 8.0], 1% NP‐40, 0.1% sodium deoxycholate, and 0.1% sodium dodecyl sulfate [SDS]) and agitated for 30 min. The cell debris was pelleted by centrifugation (5 min, 15,000*g*) and the supernatant collected. Equal volumes of protein samples were processed for denaturing gel electrophoresis and immunoblotting, using 6–14% SDS/polyacrylamide gel electrophoresis gels; Mini‐Protean Tetra Gel (Bio‐Rad) and Mini trans Blot Cell (Bio‐Rad), and proteins were blotted onto nitrocellulose membranes. Membranes were blocked in TBST (Tris Base 20 mM, NaCl 135 mM, Tween‐20 0.1%, pH 7.6) with 5% skim milk powder for 1 hr at room temperature. The membranes were then incubated overnight with anti‐alpha‐cardiac Actin Polyclonal Antibody (PA5‐21396; Invitrogen, dilution 1:500) or anti‐GAPDH (sc‐32233; dilution 1:500; Santa Cruz Biotechnology), then incubated for 1 hr with horseradish peroxidase‐conjugated secondary antibody, anti‐mouse (G21040; dilution 1:1,000; Invitrogen). Protein bands were visualized by using SuperSignal West Pico PLUS (34580; Thermo Fisher Scientific) and ChemiDoc Imaging System (Bio‐Rad). Resulting protein bands intensities were quantified with Fiji distribution of ImageJ (National Institutes of Health, Bethesda, MD).

### 2.6 Analysis of actin polymerization by cytofluorometry and confocal microscopy

The organization and quantification of polymerized actin (F‐actin) were assessed on cell cultures initiated by seeding 2 × 10^5^ cell/ml in SFM and culturing for 3 days at 37°C with 5% CO_2_. For microscopy analysis, cells were plated on coverslips placed in a 24‐well plate, fixed with 3.7% formaldehyde in PBS for 10 min and then permeabilized with 0.1% Triton X‐100 in PBS for 30 min. Samples were blocked with 2% bovine serum albumin in PBS for 1 hr and the F‐actin was stained with Alexa Fluor 647‐phalloidin (REF A22287; Thermo Fisher Scientific) at 1:100 dilution for 20 min. Coverslips were then mounted on glass slides in antifade solution containing 4′,6‐diamidino‐2‐phenylindole (SouthernBiotech REF.0100‐01). Image acquisition and signal analysis were performed on a series of Z stack pictures acquired using a confocal microscope LSM800 (ZEISS; ×40, 1.3 N.A. oil Plan‐Apochromat objective, with z‐steps of 0.5 μm). Pictures were then processed using a threshold, make binary and skeletonize functions of the ImageJ software (a Java‐based image processing program kindly provided by the National Institutes of Health).

For the analysis of actin polymerization and cell sorting by cytofluorometry, three aliquots of 10^6^ cells were collected from each culture, and the cells were resuspended in fresh media supplemented by 200 nM of SiR‐Actin (CY‐SC001; Spirochrome) and incubated for 4 hr at 37°C, 5% CO_2_. Cell fluorescence was then analyzed by cytofluorometry on a fluorescence‐activated cell sorting (BD FACS Aria II; BD Biosciences, San Jose, CA), and cells were sorted depending on their level of fluorescence (Abs 652 nm, Em 674 nm; low, medium, and high fluorescence). These cell populations were expanded and maintained at 37°C, 5% CO_2_ until further analysis.

### 2.7 Statistical analysis

The results are expressed as means ± standard error of mean. Statistical analyses were performed using Student's *t*‐test, with variance equality depending on sample variance *F*‐test. Asterisks in the figure panels refer to statistical probability *p* values of less than .05, which were considered as statistically significant.

## 3 RESULTS AND DISCUSSION

### 3.1 Effects of cytoskeleton‐related genes on therapeutic protein secretion by CHO cells

**Figure 1:**
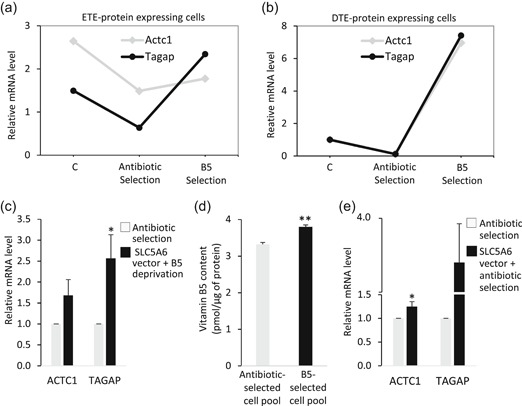
Expression of the ACTC1 and TAGAP genes following vitamin B5 selection. (a and b) Transcriptomic RNA sequencing (RNA‐seq) analyses of ACTC1 and TAGAP mRNA levels, comparing nontransfected nonselected parental control cells (C) with transfected cell pools submitted to antibiotic selection or to B5 selection and expressing trastuzumab (ETE; Panel a) and with cell lines expressing interferon β (DTE; Panel b). Transfected cells were cultured in nonselective complete culture medium, and total mRNA was isolated and submitted to high‐throughput sequencing to identify genes upregulated in cell populations submitted to the B5 selection process. The relative mRNA levels correspond to normalized read counts from RNA‐seq analyses. (c) Effect of SLC5A6 overexpression and selection by B5 deprivation on ACTC1 and TAGAP gene expression. Parental CHO cells were cotransfected with the Tras or GFP expression plasmid, and with the puromycin resistance and *SLC5A6* expression vectors, after which the cultures were selected either in the presence of puromycin (antibiotic selection), or in B5‐deficient medium (B5 deprivation), respectively. The resulting selected cell pools were transferred to a nonselective culture medium followed by the quantification of ACTC1 and TAGAP mRNAs by RT‐qPCR. mRNA levels of cells selected by B5 deprivation were normalized to those of antibiotic‐selected cells. (d) The vitamin B5 content of cells transfected and selected as described for Panel C was measured by LC–MS after 6 days of batch culture. (e) Comparison of the ACTC1 and TAGAP mRNA levels of cell pools transfected with the antibiotic resistance gene without or with the SLC5A6 expression vector and submitted to antibiotic selection. Relative mRNA levels were determined by RT‐qPCR and normalized to those of antibiotic‐resistant cells. Data are mean ± *SEM* of 3–5 biological replicates. *p ≤ .05; **p ≤ .02 with respect to antibiotic selection (*t*‐test; one tail). CHO, Chinese hamster ovary; DTE, difficult‐to‐express; ETE, easy‐to‐express; LC–MS, liquid chromatography–mass spectrometry; mRNA, messenger RNA; RT‐qPCR, real‐time quantitative polymerase chain reaction; *SEM*, standard error of mean

We recently devised a metabolic selection method whereas polyclonal cell pools and monoclonal cell lines mediating very high transgene expression levels can be more reliably isolated using a vitamin B5 deprivation method (Pourcel et al., 2020). Using this approach, CHO cells were cotransfected with expression vectors encoding an “easy‐to‐express” (ETE) trastuzumab or a “difficult‐to‐express” (DTE) infliximab or Enbrel therapeutic protein, together with the vitamin B5 transporter SLC5A6 or with an antibiotic resistance gene as a control. Cells were then selected for their aptitude to survive in a B5‐deficient culture medium or for antibiotic resistance, respectively, and differentially expressed cellular genes were identified by RNA sequencing. After antibiotic selection, the expression of both ACTC1 and TAGAP was lower than in nontransfected cells, while they were increased after B5 selection (Figure 1a,b). The increase of TAGAP expression following SLC5A6 expression and vitamin B5 starvation was validated using three independent recombinant CHO cell pools isolated using either antibiotic or B5 selections (Figure 1c).

Gene induction after B5 selection may be caused either by B5 starvation occurring during the selective process, as found in a previous study (Pourcel et al., 2020), by the overexpression of SLC5A6 itself, as it mediates higher vitamin B5 intake into the cell (Figure 1d), or by a combination of both effects. B5 is an essential cofactor for acetyl CoA, a key element in central metabolism and energy metabolism, which could be linked to cytoskeleton regulation. To distinguish between these possibilities, cell pools overexpressing the SLC5A6 transporter were generated without any B5 deprivation, which indicated that increased SLC5A6 expression suffices to upregulate significantly the ACTC1 gene, whereas a nonsignificant increase of TAGAP expression was noted (Figure 1e). Therefore, the B5 selection process might activate ACTC1 gene expression by the increased B5 intracellular import mediated by SLC5A6 overexpression, whereas a significant increase of TAGAP expression required a combination of both SLC5A6 overexpression and B5 starvation. Interestingly, we also observed that TAGAP overexpression increased ACTC1 mRNA and protein accumulation (Figure S1), suggesting that the increased ACTC1 expression resulting from the B5 selection process may result in part from the upregulation of TAGAP.

**Figure 2:**
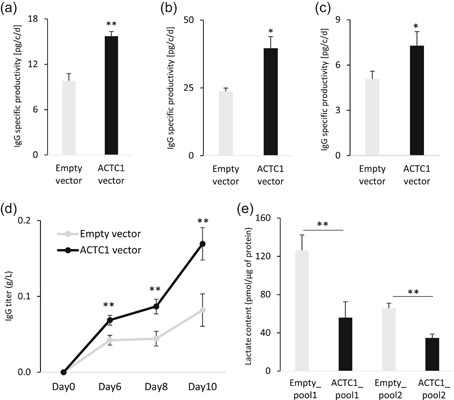
DTE recombinant protein production in cells overexpressing ACTC1. (a–c) An antibiotic‐selected IgG expressing cell line was stably retransfected with the ACTC1 or with an empty expression vector, and the IgG specific productivity of the resulting stable cell pools was measured following selection for resistance to another antibiotic. The specific productivities of the Enbrel Fc‐fusion (Panel a), the bevacizumab IgG1 (Panel b), and the infliximab IgG1 (Panel c) are represented as picograms of secreted IgG per cell and per day, as average values ± *SEM* of three replicates. (d) The levels of the infliximab IgG of cells analyzed in Panel C were assessed in fed‐batch culture conditions over 3 days in nonselective medium, where the titers of the IgG released in the cell culture medium represent the average ± *SEM* of three biological replicates. (e) The lactate content of two independent cell pools of an infliximab‐expressing cell line retransfected with the ACTC1 expression vector or with the empty vector, was measured after 3 days of a batch culture using LC–MS assays. Lactate concentrations represent mean values ± *SEM* from three technical replicates. *p ≤ .05 and **p ≤ .02 with respect to empty vector (*t*‐test; two tails). DTE, difficult‐to‐express; IgG, immunoglobulin gamma; LC–MS, liquid chromatography–mass spectrometry; *SEM*, standard error of mean

The effect of ACTC1 overexpression on recombinant protein expression was assessed on antibiotic‐selected cell lines expressing several DTE proteins, such as the Enbrel Fc‐fusion, the bevacizumab or infliximab IgG1, as well as on a cell line expressing the ETE trastuzumab immunoglobulin. These cell lines were retransfected with the ACTC1 CDS together with another antibiotic selection gene, followed by the selection of antibiotic‐resistant cells overexpressing ACTC1 (Figure S2a–c). The specific productivity of the resulting cell pools was then evaluated in batch culture conditions after 3–4 days, indicating a positive effect of ACTC1 high‐level expression on the production of the DTE proteins by CHO cells (Figure 2a–c). Further analysis of infliximab‐producing ACTC1‐overexpressing cell pools showed a significantly increased IgG titer after 10 days of fed‐batch culture (Figure 2d). A positive effect of TAGAP on recombinant protein expression was also observed (see accompanying paper, Berger et al., 2020).

**Figure 3:**
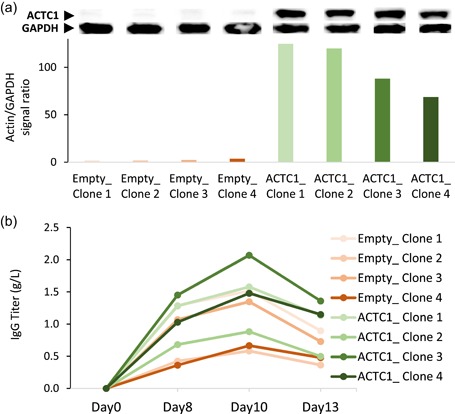
Characterization of the productivity of ACTC1‐overexpressing cells. A trastuzumab‐expressing line was stably retransfected with the CHO ACTC1 or with an empty expression vector, and cell clones were isolated for further analysis. (a) Immunoblots of total protein extracts labeled with ACTC1 or GAPDH mouse antibodies. The histogram shows the ratio of the ACTC1 signal relative to that of GAPDH, as assessed using the ImageJ. Ponceau red‐stained membranes are shown in Figure S3a. (b) Secreted IgG titers in culture supernatants were assessed by double sandwich ELISA over 13 days of fed‐batch cultures. CHO, Chinese hamster ovary; ELISA, enzyme‐linked immunosorbent assay; IgG, immunoglobulin gamma [Color figure can be viewed at wileyonlinelibrary.com]

We next analyzed individual clones overexpressing ACTC1. To do so, a trastuzumab‐producing clone was retransfected with the ACTC1 or empty expression vector, and single colonies were picked using a Clonepix device. Eight clones transfected with the empty vector and 24 ACTC1‐expressing clones were validated for ACTC1 transcript accumulation, among which four control clones and four ACTC1 high expressor clones were randomly picked for further analysis (Figure S3a). ACTC1 protein overexpression was validated by western blot (Figures 3a and S3b). Among the four ACTC1‐overexpressing clones, three showed the highest IgG titers after 13 days of fed‐batch culture as compared with the empty vector clones (Figures 3b and S3c).

To determine if the increased therapeutic protein secretion elicited by ACTC1 overexpression may result from cellular metabolic alterations, we measured primary metabolism markers by MS analysis of pools of ACTC1‐overexpressing cells. Notably, we assessed the accumulation of lactate, a toxic by‐product of the early steps of glycolysis, which has been well documented as a bottleneck for therapeutic protein production (Lao & Toth, 1997). This revealed a strong reduction of lactate accumulation by ACTC1‐overexpressing cells after 3 days in batch culture when compared with control cells (Figure 2e). Overall, we, therefore, concluded that ACTC1 gene overexpression significantly improved the secretion of various therapeutic proteins and that this effect may be linked to a decrease in the accumulation of the toxic lactate metabolic by‐product.

### 3.2 Implication of actin filament organization and level in recombinant protein secretion

**Figure 4:**
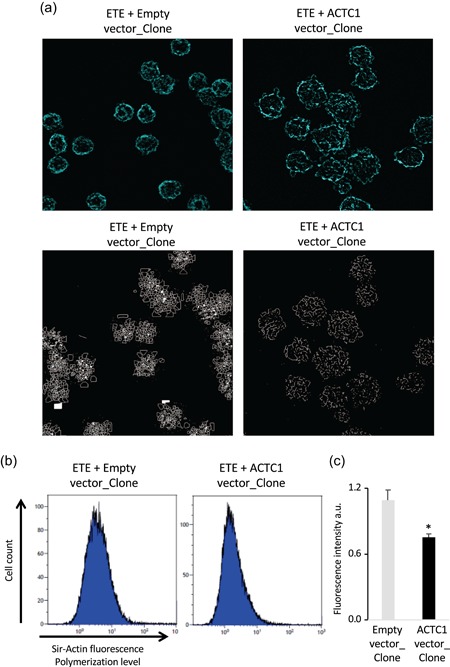
Actin organization in ETE clones. (a) Phalloidin staining of a traztuzumab IgG‐expressing cell line overexpressing ACTC1 (ACTC1_Clone, right panels), or from a control cell line transfected with the empty expression vector (Empty_Clone, left panels). The upper panels depict a picture of the cell's fluorescent phalloidin staining while the lower panel is an ImageJ processed image highlighting the filament structure. (b) Sir‐actin fluorescent F‐actin staining of trastuzumab‐expressing clone two cells overexpressing ACTC1 (ETE + ACTC1_Clone) or transfected with the empty expression vector (ETE + Empty_Clone), as assessed using cytofluorometry. Unstained cells were used as negative controls. (c) Mean fluorescent signal of Sir‐actin staining from flow cytometry analyses. A total of 2 × 10^4^ cells were analyzed per condition. Data illustrated on the graph represent the mean ± *SEM* from the assay of four independent clones. *p < .05 (*t*‐test; two tails; unequal variance). ETE, easy‐to‐express; IgG, immunoglobulin gamma; *SEM*,  standard error of mean [Color figure can be viewed at wileyonlinelibrary.com]

We next assessed whether the actin polymerization status may be affected by ACTC1 overexpression. Phaloidin staining of F‐actin was performed on independent cell lines expressing ETE proteins, revealing structural differences. Negative control cells expressing only the ETE proteins displayed round actin filament structures, as observed for the untransfected parental CHO cells (Figures 4a, S4, and S5; Movie S1). However, sharp actin structures appeared when ACTC1 was overexpressed, and cells displayed a somewhat bloated shape (Figures 4a, S4, and S5; Movie S2). F‐actin staining was abolished when cytochalasin, an actin polymerization inhibitor, was added (Figure S5).

To further assess actin polymerization levels, we relied on SiR‐actin staining, which specifically binds to F‐actin (Lukinavičius et al., 2014), yielding fluorescence level of stained cells that are proportional to actin polymerization. Comparison of SiR‐actin‐staining of the ACTC1 and trastuzumab‐expressing clones relative to control clones revealed higher fluorescence in the control clones than in the ACTC1‐overexpressing ones, implying that actin polymerization levels are reduced by ACTC1 overexpression (Figures 4b,c and S6).

**Figure 5:**
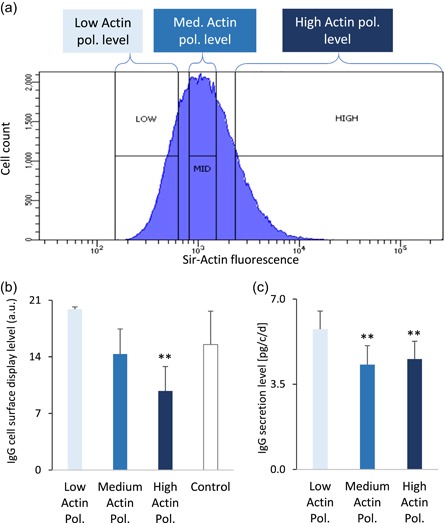
Sorting of therapeutic protein‐producing cell pools according to their F‐actin polymerization level. Representative histograms of flow cytometry analyses of a trastuzumab‐expressing cell pool treated by SiR‐actin staining. Unstained control and other analysis are depicted in Figure S4. A total of 5 × 10^5^ cells were analyzed per acquisition, among which 0.4–1.4 × 10^5^ cells were sorted by cytofluorometry according to their low, medium, or high actin polymerization (pol.) levels, as depicted. (b) Selected cells were transferred to an antibiotic‐containing culture medium followed by the analysis of IgG cell surface display by immunofluorescence staining cytofluorometry. (c) IgG secretion assays of the sorted cells of Panel B. Histograms represent the average values ± *SEM* from six cell pools. **p ≤ .02 (*t*‐test; two tails; paired) relative to the low actin polymerization category. IgG, immunoglobulin gamma; *SEM,* standard error of mean [Color figure can be viewed at wileyonlinelibrary.com]

To assess whether actin polymerization may affect recombinant protein expression, two independent CHO cell pools expressing the trastuzumab protein, but not submitted to an ACTC1 vector transfection, were stained with SiR‐actin. Stained cells were then sorted in three independent cell batches according to their low, medium, or high fluorescence level (Figures 5a and S7), to obtain six‐cell pools for each fluorescence level. The IgG secretion level and IgG specific productivity of cells displaying low, medium, and high levels of polymerized actin was then assessed (Figure 5b,c). High SiR‐actin staining cells showed a significantly lower IgG expression levels than cells displaying low SiR‐actin staining, thus supporting the conclusion that cells with lower actin polymerization levels mediate higher recombinant protein secretion, even without ACTC1 overexpression.

## 4 CONCLUSION

In this study, we showed that overexpressing *ACTC1* actin gene, known to be involved in cardiac muscle alpha‐actin synthesis, also acts to improve ETE and DTE therapeutic protein expression and secretion by CHO cells. We observed that the increase of ACTC1 levels was accompanied with a decrease in overall actin polymerization, implying that the organization of the cytoskeleton controls or affects the expression or the secretion of the therapeutic proteins. To support this observation, we showed that CHO cell pools with spontaneously decreased actin polymerization level secrete significantly higher levels of the recombinant protein. Since the augmented release of therapeutic proteins by actin‐overexpressing cells was not accompanied by increased IgG light and heavy chain mRNA (data not shown), we conclude that this actin effect is posttranscriptional.

We hypothesize that ACTC1 overexpression accumulates an excess of actin monomers, which may disturb intracellular balance with G/F‐actin and thereby cause the observed decrease of the F‐actin polymeric forms and altered organization of the cytoskeleton. An interplay of actin dynamics and gene expression has already been proposed in mammalian cells. For instance, it was found that the treatment of primary murine cells with chemical agents provoking F‐actin disruption ellicited a global inhibition of translation and protein synthesis and that this activated the cellular stress response (Silva, Sattlegger, & Castilho, 2016). Here, we show that a decrease of actin polymerization, either spontaneous or elicited by ACTC1 overexpression, rather mediated an increase of recombinant protein expression by CHO cells and that this did not impair cell division or viability. Further work will be required to fully understand this phenomenon, but we infer that F‐actin depolymerization may provoke a turnover of actin assembly that may enhance vesicular and protein trafficking. For instance, colifin is an actin‐depolymerizing protein that induces actin reorganization, thereby promoting the exocytosis of small molecules and vesicular trafficking (Birkenfeld, Kartmann, Betz, & Roth, 2001). Similarly, CHO suspension cells selected for lower levels of polymerized actin may display higher cytoskeletal reorganization, which in turn may improve recombinant protein secretion. However, another favorable effect of ACTC1 overexpression is the resulting decrease in the accumulation of the cell‐toxic lactate by‐product of early glycolysis. An interplay of the cytoskeleton with lactate accumulation was suggested by a report showing that cytoskeleton perturbation can inhibit the lactate transporter and import by oocytes (Tosco, Faelli, Gastaldi, Paulmichl, & Orsenigo, 2008). These observations suggest that CHO cell actin depolymerization might prevent the accumulation of toxic intracellular lactate concentrations.

We, therefore, conclude that several mechanisms may well explain the positive effect of actin overexpression on protein production by CHO cells, which may pertain both to the basic metabolism of CHO cell and energy production by glycolysis, as well as by a potential activation of protein secretion. Further work will be required to determine the contribution of these mechanisms on recombinant protein accumulation by cultured CHO cells. In any case, this study shows that ACTC1 overexpression and/or the assay for spontaneous alterations in F‐actin polymerization using SiR‐actin staining and cell sorting can both be used to facilitate the isolation of high expressor CHO cells from stable cell pools.

As for ACTC1, TAGAP overexpression improved recombinant protein secretion. TAGAP enzymatic activity was showed to regulate the organization of actomyosin fibril, to release integrin‐mediated adhesion in thymocytes, which is necessary for directed‐thymocyte migration from the cortex to the medulla (Duke‐Cohan et al., 2018). In suspension CHO cells, TAGAP could function as a mediator for intracellular cytoskeleton signal to cell surface integrins, hence improving cell proliferation, viability, and adaptation to suspension.

Overall, we conclude that cytoskeletal proteins and the modulation of the cytoskeletal organizations may be used to improve protein production for biotechnological purposes. It will be interesting to assess the ability of actin monomer‐binding agents that mediate transient actin depolymerization to modulate protein translation and secretion in CHO cells, and whether this may provide easy approaches towards increasing the therapeutic production capacity of these cells.

## CONFLICT OF INTERESTS

G. A., V. L., A. R., I. B., and P. A. G. are employed by—and N. M. is a consultant of—Selexis SA, a company that generates Chinese hamster ovary cell clones expressing therapeutic proteins. Selexis SA did not contribute to or influence the writing of the manuscript.

## AUTHOR CONTRIBUTIONS

L. P. designed the project; L. P., V. L., G. A., F. B., and A. R. performed the experiments; L. P. and N. M. analyzed the data and wrote the paper.

## Supporting information

Supplementary information

Supplementary information

Supplementary information
