# Supplementary material for: Influence of cytoskeleton organization on recombinant protein expression by CHO cells
Source: Biotechnol Bioeng. 2020 Feb 23;117(4):1117–26. doi: 10.1002/bit.27277 (PMC7079171; doi:10.1002/bit.27277)
Supplement: Supplementary file 1 — Supplementary information [file BIT-117-1117-s001.PDF]

FIG. S1

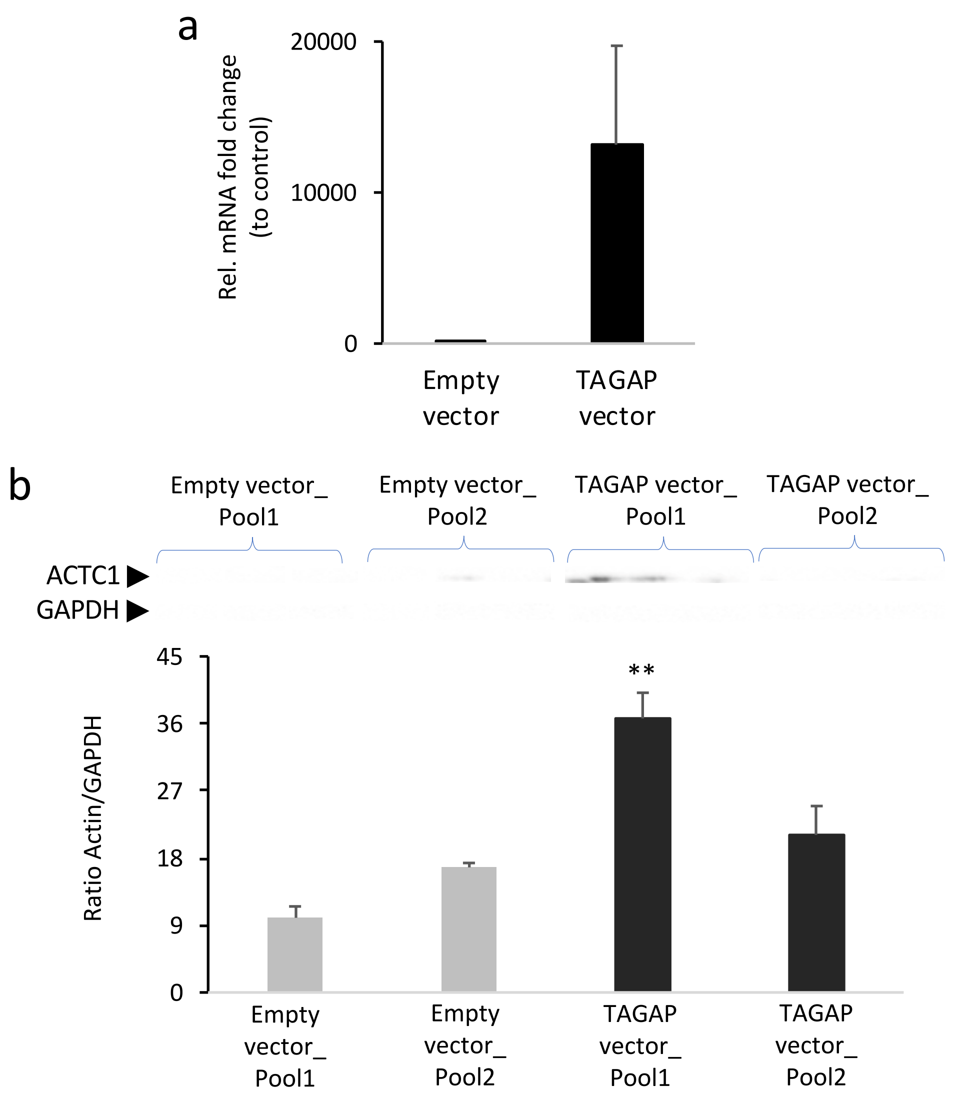

FIGURE S1. **ACTC1 levels in ETE-producing cells overexpressing TAGAP**

A puromycin-selected cell line expressing the Trastuzumab antibody was stably re-transfected with CHO TAGAP expression vector, or with an empty vector and blasticidin resistance gene, and selected for blasticidin resistance. Resulting stable polyclonal cell pools were used to assess TAGAP relative mRNA levels by RT-qPCR **(a)**; and the ACTC1 protein levels **(b)**. Immunoblots of total protein extracts probed with ACTC1 or GAPDH mouse antibodies. The ratio of the ACTC1 signal was normalized to that of GAPDH, as quantified by ImageJ. Data represent the mean fluorescence ±SEM of 3 replicates. ***P*≤0.02 with respect to cells transfected with the empty vector (t-test; 2 tails).

FIG. S2

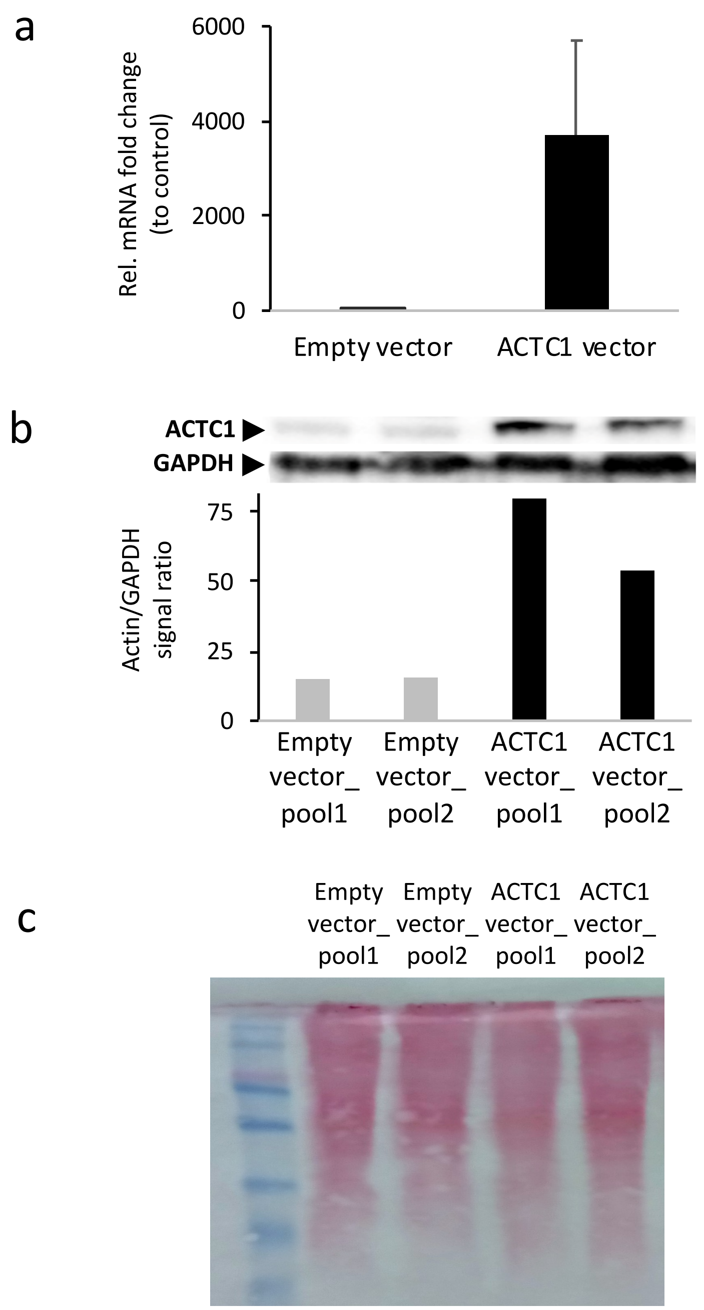

FIGURE S2. **Overexpression of ACTC1 in recombinant protein-producing cells**
(a) A puromycin-selected cell line expressing the infliximab antibody was stably re-transfected with CHO ACTC1 expression vector, or with an empty vector and blasticidin resistance gene, and selected with blasticidin resistance. The resulting stable cell pools were used to quantify the relative mRNA levels of ACTC1 by RT-qPCR. **(b)** Immunoblots of total protein extracts from the cell pools of panel B, probed with ACTC1 or GAPDH mouse antibodies. The ratio of the signal for ACTC1 relative to that of GAPDH was quantified using ImageJ. **(c)** Red Ponceau staining of total protein of the immunoblot membranes of panel C. Data represent the mean values ±SEM of 3 replicates.

FIG. S3

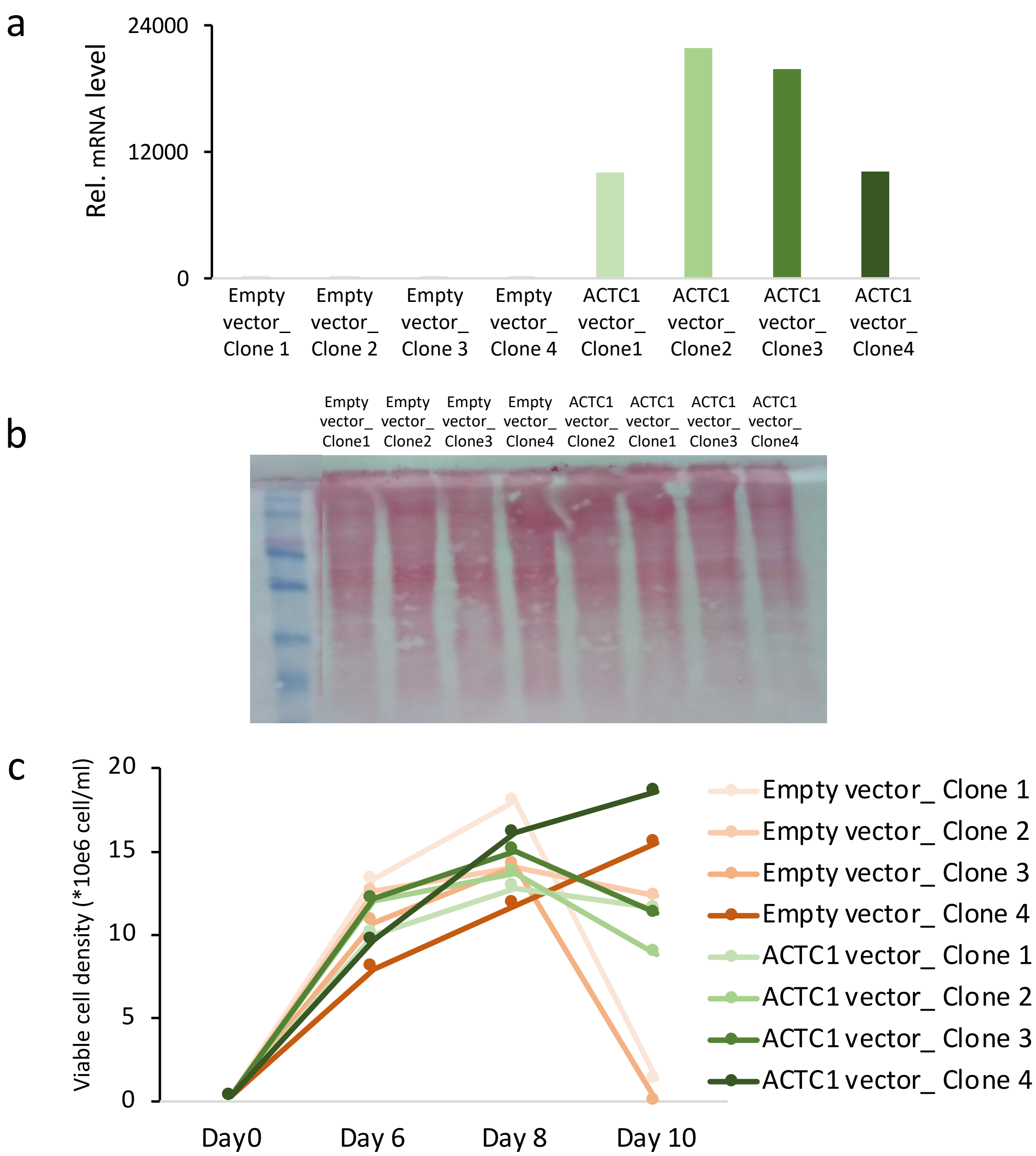

FIGURE S3. Characterization of ACTC1-overexpressing cells

A Trastuzumab-expressing CHO cell line was stably re-transfected with an antibiotic resistance plasmid, together with the CHO ACTC1 expression vector or with the empty expression vector. Stably transfected antibiotic-resistant cells were then selected, from which clones were isolated for further analysis. **(a)** Quantification of ACTC1 relative mRNA levels, as determined by RT-qPCR. **(b)** Red Ponceau staining of total protein of the immunoblot membrane of Figure 3a. **(c)** Viable cell density of the clones over 10 days of the fed-batch cultures performed in Fig. 3b.

FIG. S4

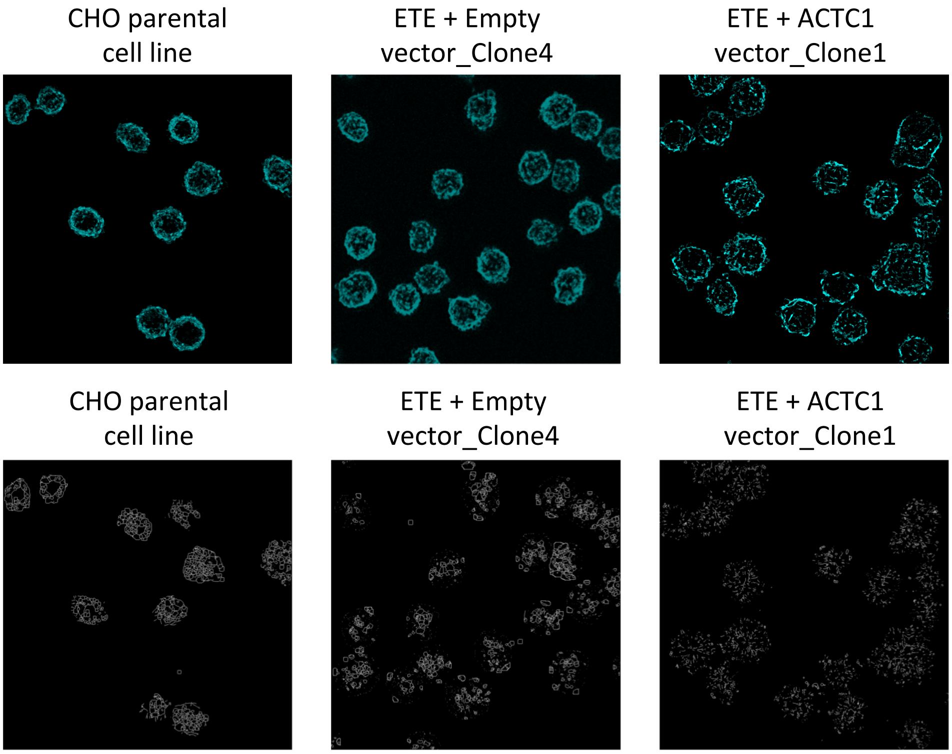

FIGURE S4. Actin filament organization in recombinant clones overexpressing ACTC1
Phalloidin staining of clones from a Traztuzumab IgG-expressing cell line overexpressing ACTC1 (ETE + ACTC1 vector_Clone 1, right panel), or transfected with an empty expression vector (ETE + Empty vector_Clone 4, left panel). The upper panels depict a picture of the cells fluorescent phalloidin staining while the lower panel is an ImageJ processed image highlighting the filament structure.

FIG. S5

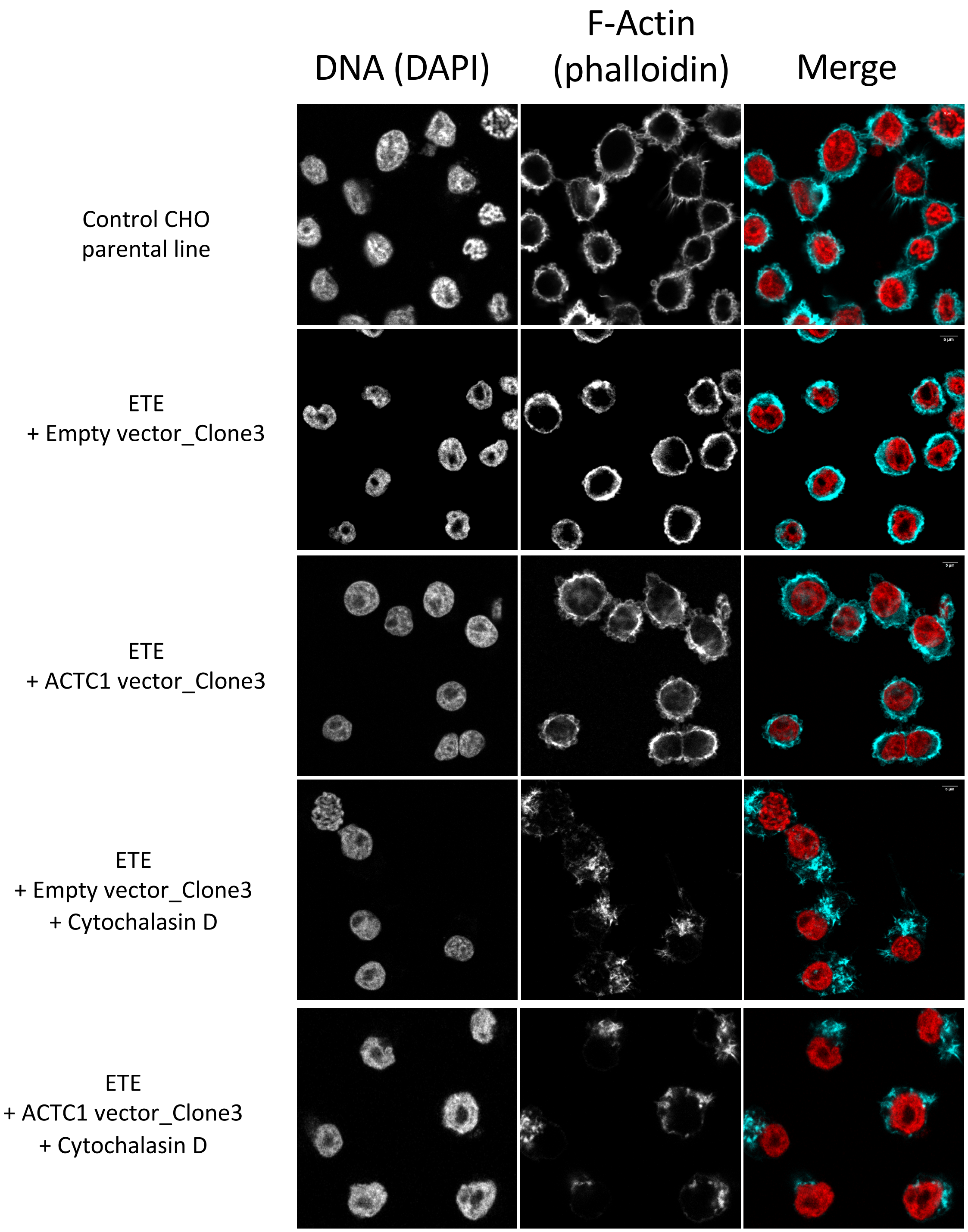

FIGURE S5. Actin filament organization and staining specificity
DAPI staining (left panel); phalloidin fluorescent staining (middle panel) and merged stainings (right panel) of CHO parental cell line, a Traztuzumab clone expressing an empty vector (ETE + Empty_Clone3) or a Traztuzumab clone overexpressing the ACTC1 expression vector (ETE + ACTC1_Clone3), without or with cytochalasin D.

FIG. S6

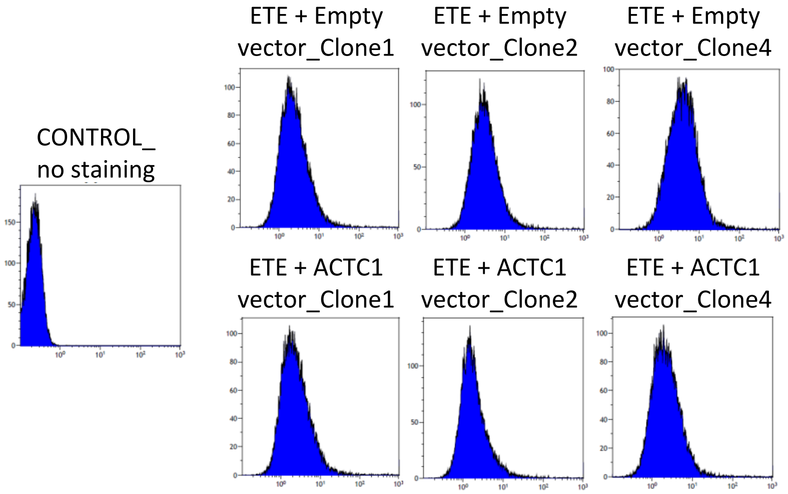

FIGURE S6. **Actin polymerization levels in ETE clones**

Sir-actin fluorescent histograms of F-actin on cells from all trastuzumab clones tested, overexpressing ACTC1 (ETE + ACTC1 vector_Clones), or from control clones transfected with the empty expression vector (ETE + Empty vector_Clones), obtained from flow cytometry. Unstained cells were used as negative controls.

FIG. S7

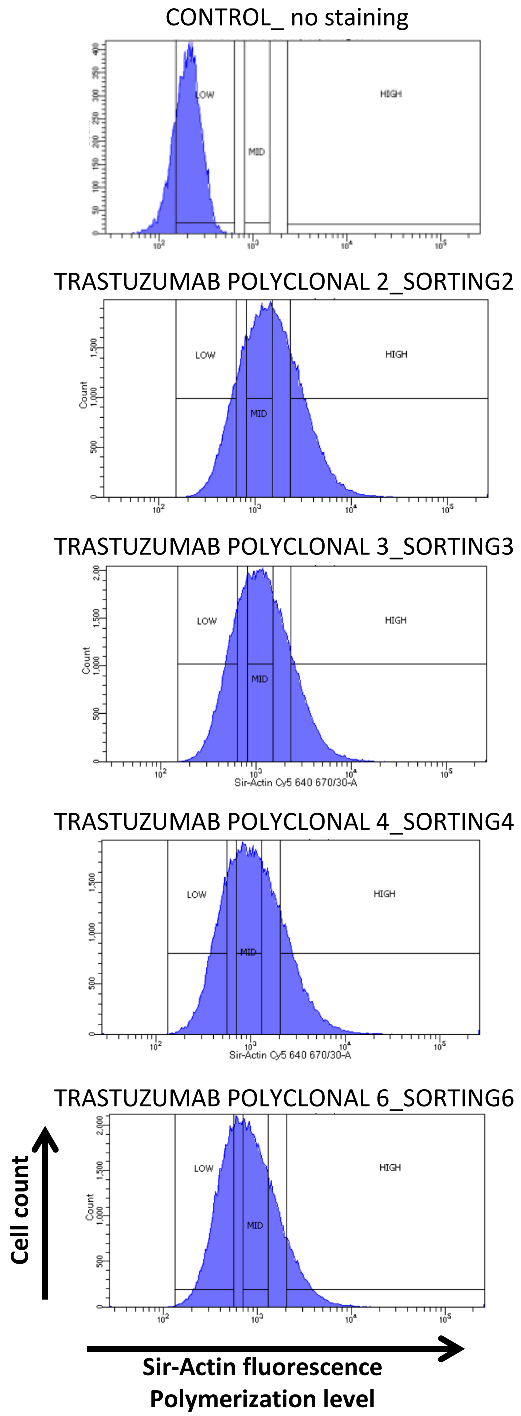

FIGURE S7. **Sorting of Trastuzumab-expressing cell pools according to their actin polymerization level**

Representative histograms of flow cytometry analyses of a trastuzumab polyclonal population treated by Sir-actin staining, as described in the legend to Figure 5. The histogram at the top corresponds to unstained control cells and the ones below represent cytometry analysis of independent Sir-actin stained cell pools.
